# Value of estrogen pretreatment in patients with diminished ovarian reserve and elevated FSH on a line antagonist regimen: a retrospective controlled study

**DOI:** 10.1186/s13048-024-01415-2

**Published:** 2024-05-27

**Authors:** Lin Lin, Guoyong Chen, Yun Liu

**Affiliations:** 1 Center for Reproductive Medicine, Dongfang Hospital, Xiamen University (900TH Hospital of Joint Logistics Support Force), Fuzhou, China; 2 Center for Reproductive Medicine, Fuzong Clinical College, Fujian Medical University (900TH Hospital of Joint Logistics Support Force), West Second Ring North Road, Fuzhou, 350025 Fujian Province China

**Keywords:** Diminished ovarian reserve, High basal FSH, Antagonist regimens, Estrogen pretreatment

## Abstract

**Background:**

The key to enhancing the efficacy of antagonistic regimens in pregnancy is to better synchronize follicular growth during cycles of controlled ovarian stimulation (COS), especially in patients with diminished ovarian reserve (DOR). During in vitro fertilization-embryo transfer (IVF-ET) treatment, luteal phase estrogen pretreatment may enhance follicular development synchronization and yield of mature oocytes. However, the effect of estrogen pretreatment in DOR patients with elevated basal follicle-stimulating hormone (FSH) levels has not been well studied.

**Methods:**

We retrospectively analyzed the clinical data of patients with elevated basal FSH levels and DOR (401 cycles) who underwent IVF/intracytoplasmic monosperm injection (ICSI)-assisted conception. Both groups were treated with a flexible gonadotropin-releasing hormone (GnRH) antagonist regimen and were further divided into two groups according to whether they received luteal estrogen pretreatment. There were 79 patients in the estrogen pretreatment group and 322 patients in the control group. On the second day of the menstrual cycle, gonadotropin (Gn) stimulation of the ovaries was initiated. The general characteristics, clinical, biological parameters and outcomes of the two groups were compared.

**Results:**

The basic profiles of the two groups were similar (*P* > 0.05). More patients in the pretreatment group showed FSH rebound after gonadotropin (Gn) initiation, resulting in a significantly higher number of Gn days and total Gn than those in the control group (*P* < 0.05). There was no statistically significant difference in the number of days of antagonist use, follicle output rate (FORT), number of metaphase II(MII)eggs obtained, number of Two pronuclei (2PN) fertilized, number of D_3_ quality embryos, blastocyst formation rate, fresh embryo clinical pregnancy rate, cumulative pregnancy rate, and non-transferable embryo rate between the two groups (*P* > 0.05).

**Conclusions:**

The use of luteal phase estrogen pretreatment in patients with elevated basal FSH combined with DOR resulted in high FSH levels after the release of negative feedback, which was detrimental to early follicular growth, did not increase the follicular output rate, may have increased the use and duration of controlled ovarian stimulation drugs, and did not increase the number of eggs gained or improve clinical outcomes.

## Background

An increasing number of infertile patients with diminished ovarian reserve (DOR) are seeking in vitro fertilization–embryo transfer (IVF–ET) fertility treatment. These patients are prone to poor ovarian response (POR) during ovarian stimulation compared to patients with normal ovarian reserve, resulting in fewer eggs being obtained and further reducing pregnancy rates. Gonadotropin-releasing hormone (GnRH) antagonist regimens are recommended for controlled ovarian stimulation (COS) cycles in patients with POR because of their short stimulation time and low cost [1]. Improving the synchronization of follicular development in COS cycles is the key to improving pregnancy outcomes with antagonistic regimens. Luteal Phase E_2_ reduces the size and improves the homogeneity of early antral follicles. This approach may help to synchronize follicular development in COS cycles [2, 3]. However, luteal phase estradiol pretreatment with a GnRH antagonist regimen did not affect reproductive outcomes in a normally ovarian-responsive population. It may be because patients with normal ovarian function can obtain enough oocytes during COS to counteract the negative effects of follicular desynchronization in the GnRH antagonist regimen [4]. Significantly elevated basal follicle-stimulating hormone (FSH) levels suggest severe DOR function and possibly even ovarian failure. The number of eggs and embryos decreases in individuals with elevated basal FSH levels [5]. Whether estrogen pretreatment can improve the number of obtained eggs and embryos in patients with DOR and elevated basal FSH has not been well studied. Investigating whether luteal phase estrogen pretreatment can help such patients produce more oocytes on antagonist regimens and thus improve pregnancy outcomes is a pressing challenge in the field of reproductive fertility. In this study, we retrospectively analyzed data on IVF cycles in patients with elevated basal FSH levels combined with DOR to investigate the effectiveness and necessity of estrogen pretreatment in the application of antagonist regimens.

## Methods

### Patients and study design

This study was approved by the Ethics Committee of 900TH Hospital of the Joint Logistics Support Force. Informed consent was obtained from all participating couples. All patient information was anonymized and strictly confidential.

We retrospectively analyzed the clinical data of patients with elevated basal FSH levels combined with DOR (401 cycles) who underwent IVF/intracytoplasmic monosperm injection (ICSI)-assisted conception treatment at the 900TH Hospital of Joint Logistics Support Force Reproductive Center, Fuzhou, China, from January 2019 to October 2022. Inclusion criteria included (1) age < 45 years; (2) basic FSH ≥ 10 U/L; in addition to at least two of the following three criteria: (3)vaginal ultrasound suggestive of bilateral ovarian antral follicle count (AFC) ≤ 7 [6, 7]; (4)anti-mullerian hormone (AMH) < 1.1 ng/ml [6, 7]; (5)previous cycles with low ovarian response and ≤ 3 eggs obtained by conventional protocol [6]. Exclusion criteria included: (1) chromosomal abnormalities in both or one of the couple; (2) coexistence of relevant diseases affecting IVF pregnancy outcome, such as severe adenomyosis, untreated hydrosalpinx, untreated endometrial lesions, or uterine fibroids ≥ 4 cm; and (3) patients with endocrine metabolic diseases such as polycystic ovary syndrome (PCOS). There are no effective treatments to improve outcomes in these severe cases of DOR. E_2_ pretreatment may be able to increase oocyte production and improve outcomes. A total of 401 cycles were included and divided into two groups according to whether estrogen pretreatment was performed during the luteal phase: there were 79 patients in the estrogen pretreatment group and 322 patients in the control group (i.e., no estrogen pretreatment). Early patients got estrogen pretreatment (source of 79 experimental cohorts) because we thought that pretreatment with an antagonist regimen could enhance the synchronization of follicular clusters. On the other hand, we discovered that FSH rebound was common in patients with basal FSH ≥ 10 U/L in addition to DOR. Consequently, estrogen therapy was eventually stopped in these individuals (source of 322 controls). The treatment parameters employed by the medical staff were uniform.

### Pretreatment schemes

All patients received 17β-estradiol (Fentanyl Red Tablets, Abbott Laboratories, Netherlands). Ovulation was monitored one cycle prior to the antagonist regimen for ovulation. The objective of the ultrasound examinations was to evaluate the number and sizes of early antral follicles. 2 mg of oral 17β-estradiol was taken twice daily, starting 7 to 8 days after ovulation and discontinued after the second day of menstruation. The patient was pretreated with estrogen and did not have any washout period prior to initiation of gonadotropins. A potential disadvantage of considering any washout period is the gradual release of endogenous FSH prior to initiation of FSH, which may compromise the coordination required for antral follicle size. All patients had venous blood draws completed between 7am and 7:15am.

### COH and IVF/ICSI–ET protocols

On the second day of menstruation, gonadotropin (Gn), including recombinant FSH (Prilosec, Merzadone, Germany; Gonafine, Merck Serono, Switzerland) or urinary-derived FSH (Lishenbao, Zhuhai Lizhu Pharmaceuticals) was administered at a dose of 150–300 IU/d, adjusted according to the responsiveness of the follicles. When the maximum follicle diameter reached 12–13 mm, the E_2_ level exceeded 400 pg/mL, and/or luteinizing hormone (LH) > 5 U/L, 0.25 mg/day cetrorelix acetate (Cetrotide, Merck Serono, Switzerland) was administered until HCG day. When the diameter of the dominant follicle reached 18 mm, HCG was injected at 6,000–10,000 U that night, and eggs were retrieved by vaginal ultrasound-guided puncture 36–38 h later.

The obtained oocytes were subjected to conventional IVF or ICSI in vitro fertilization, and fertilization was observed 16–18 h after insemination. High-quality embryos included normal fertilization, day 3, and day 5/6 embryos of high quality (day 3 embryos of grade 1–2, 7–9 ovoid spheres, < 20% fragmentation ; blastocysts at least at expansion stage 3 with an endocytic quality score of A or B, and day 5 trophectoderm score of A or B). On the 3rd day after egg retrieval, 1 ∼ 2 embryos with the highest grade were routinely transferred, while the rest were cultured, and blastocysts with grades of 4BC, 4CB, or higher were frozen. All embryo freezing was performed if one of the following conditions was met: progesterone (P) level on the day of HCG > 1.4 ng/ml, endometrial thickness < 6 mm, and the presence of cavity occupancy or uterine adhesions not suitable for fresh embryo transfer.

The frozen–thawed embryo transfer (FET) protocol was performed one to two menstrual cycles after egg retrieval. One blastocyst, or one to two cleavage-stage embryos were transferred depending on the regularity of the patient’s menstrual cycle and the condition of the endometrium; a natural cycle or hormone replacement cycle was selected.

### Outcome measures

The primary outcomes were the number of high-quality embryos, clinical pregnancy rate of fresh embryo transfer, and cumulative pregnancy rate; secondary outcomes were the number of MII eggs, Gn dose, and duration. Clinical pregnancy was diagnosed by ultrasound detection of a gestational sac 2 weeks after a positive hCG test.

### Statistical analysis

The SPSS 26.0 software package (IBM Corp., Armonk, NY, USA) was used for statistical analysis. Normally distributed data were represented as mean and standard deviation (SD), and skewed data are described as the median and interquartile range (IQR). We used the chi-square test or Fisher’s exact test (when appropriate) to make statistical inferences about the qualitative data. We used the t-test or Mann–Whitney U test to compare continuous variables, as required. A probability (*P*) value < 0.05 indicated that the difference between the two groups was statistically significant.

## Results

In total, 401 patients were enrolled in this study. There were no significant differences in age, years of infertility, basal FSH, basal LH, basal estradiol (E_2_), AMH, AFC, body mass index (BMI), or cause of infertility (tubal factor, unexplained infertility, male factor, combining male and female Factors) between the two groups (Table 1).

**Table 1:** Baseline characteristics of the study population

Characteristics/Variables	Pretreatment group (*n* = 79)	Control group (*n* = 322)	*P*-value
Age (years)	37.24 ± 3.59	37.72 ± 4.61	0.314
Infertility duration (years)	4.03 ± 2.71	3.79 ± 2.51	0.457
BMI (Kg/m^2^)	21.72 ± 2.01	21.63 ± 1.98	0.706
AMH (ng/mL)	0.75 ± 0.48	0.73 ± 0.45	0.668
AFC (n)	4.65 ± 1.74	4.60 ± 1.65	0.791
Cause of infertility (%)			
Tubal factor, n (%)	52/79(65.8)	210/322(65.2)	
Unexplained infertility, n (%)	1/79(1.3)	7/322(2.2)	
Male factor, n (%)	10/79(12.7)	29/322(9.0)	
Combining male and female Factors, n (%)	14/79(17.7)	65/322(20.2)	
Three AIH failure history, n (%)	2/79(2.5)	11/322(3.4)	
Basal FSH/ (U/L)	11.14(10.24–12.27)	11.18(10.32–12.51)	0.093
Basal LH/ (U/L)	4.43 ± 2.00	4.68 ± 2.07	0.343
Basal E_2_ / (pg/mL )	45.81 ± 17.07	48.24 ± 21.84	0.253

### Ovulation promotion in the two groups

The FSH, LH, progesterone (P) levels, and mean and maximum follicle diameters of the pretreatment group on the initiation day were significantly lower than those of the control group (*P* < 0.05); E_2_ levels in the pretreatment group were significantly higher than those in the control group (*P* < 0.05); the number of follicles in the two groups on the day of initiation was not statistically different (*P* > 0.05). The FSH and LH levels in the pretreatment group on Gn5 days were higher than those in the control group (*P* < 0.05); the E_2_ level, mean follicle diameter, and maximum follicle diameter in the pretreatment group were significantly lower than those in the control group, and the difference was statistically significant (*P* < 0.05), and there was no statistically significant difference in progesterone levels and follicle numbers between the two groups (*P* > 0.05). FSH levels in the pretreatment group on the antagonist start day were higher than those in the control group (*P* < 0.05); the LH, E_2_, and P levels in both groups were not significantly different (*P* > 0.05); the number of ≥ 8 mm follicles, the mean follicle diameter and its coefficient of variation, and the maximum follicle diameter were similar in both groups (*P* > 0.05). The FSH, LH, E_2_, and P levels in both groups on the day of HCG administration were not significantly different (*P* > 0.05); the number of ≥ 14 mm follicles, mean follicle diameter and its coefficient of variation, and maximum follicle diameter were similar in the two groups (*P* > 0.05). The number of Gn days and total Gn dose in the pre-treatment group were significantly higher than those in the control group (*P* < 0.05). The Gn initiation dose, days of antagonist use, and follicle output rate (FORT) were not significantly different between the two groups (*P* > 0.05; Table 2).

**Table 2:** Outcomes of ovarian stimulation

	Pretreatment group (*n* = 79)	Control group(*n* = 322)	*P*-value
**Day 2 hormone level and ultrasound**			
FSH (mIU/l)	4.61 ± 1.74	11.81 ± 2.43	0.000
LH (mIU/l)	2.07 ± 0.96	4.71 ± 3.13	0.000
E_2_ (pg/ml)	163.64 ± 68.28	49.97 ± 21.52	0.000
P (ng/ml)	0.63 ± 0.30	0.72 ± 0.38	0.023
Number of follicles (n)	4.72 ± 1.40	4.53 ± 1.68	0.343
Mean follicle diameter (mm)	3.79 ± 0.52	4.83 ± 0.77	0.000
CV^a^%	13.76	16.02	
Maximum follicle diameter (mm)	4.63 ± 0.97	6.42 ± 0.96	0.000
**Gn 5 (day 6) hormone level and ultrasound**			
FSH (mIU/l)	22.75 ± 5.96	15.82 ± 3.29	0.000
LH (mIU/l)	4.00 ± 2.40	2.99 ± 1.55	0.000
E_2_ (pg/ml)	93.37 ± 70.89	202.14 ± 3.68	0.000
P (ng/ml)	0.51 ± 0.15	0.53 ± 0.13	0.120
Number of follicles (n)	3.25 ± 0.94	3.40 ± 0.92	0.203
Mean follicle diameter (mm)	6.84 ± 1.63	8.39 ± 1.73	0.000
CV (%)	23.76	20.57	
Maximum follicle diameter (mm)	8.12 ± 2.17	11.25 ± 1.70	0.000
**On antagonist start day**			
FSH (mIU/l)	17.04 ± 4.67	15.59 ± 2.54	0.015
LH (mIU/l)	3.21 ± 1.73	3.30 ± 1.44	0.634
E_2_ (pg/ml)	351.71 ± 163.51	328.71 ± 162.16	0.288
P (ng/ml)	0.51 ± 0.13	0.55 ± 0.15	0.058
Number of follicles ≥ 8 mm (n)	2.63 ± 1.01	2.55 ± 1.03	0.579
Mean follicle diameter (mm)	12.24 ± 1.44	11.97 ± 1.38	0.145
CV (%)	11.80	11.51	
Maximum follicle diameter (mm)	13.70 ± 1.57	13.91 ± 1.97	0.549
**On hCG trigger day**			
FSH (mIU/l)	15.17 ± 4.38	15.71 ± 2.94	0.554
LH (mIU/l)	3.58 ± 2.97	3.36 ± 2.50	0.680
E_2_ (pg/ml)	2449.54 ± 1209.56	2749.20 ± 1304.43	0.214
P (ng/ml)	0.54 ± 0.15	0.58 ± 0.16	0.065
Number of follicles ≥ 14 mm (n)	2.20 ± 0.94	2.04 ± 1.08	0.269
Mean follicle diameter (mm) ≥ 14 mm	17.47 ± 0.92	17.62 ± 1.20	0.244
CV (%)	5.28	6.80	
Maximum follicle diameter (mm)	18.28 ± 1.32	18.45 ± 1.31	0.319
Duration of GnRH antagonist (days)	3.37 ± 1.33	3.57 ± 1.02	0.178
Duration of Gn (days)	10.83 ± 1.98	8.48 ± 2.16	0.000
Gn initiation dose (IU)	200.63 ± 47.36	192.86 ± 41.87	0.151
Total dose of Gn (IU)	1919.29 ± 817.47	1596.45 ± 631.88	0.000
Follicle output rate ^b^ (%)	47.63 ± 27.88	47.92 ± 25.84	0.933

Note:

^a^coefficent of varience(CV) = standard deviation of mean follicle diameter/mean follicle diameter [8]

^b^Follicular output rate determined by the ratio of the preovulatory follicle (14–22 mm) count on the HCG trigger day×100/the small antral follicle (3–8 mm) count at baseline [9]

### Trends in FSH and E_2_ change during ovulation promotion

**Fig. 1:**
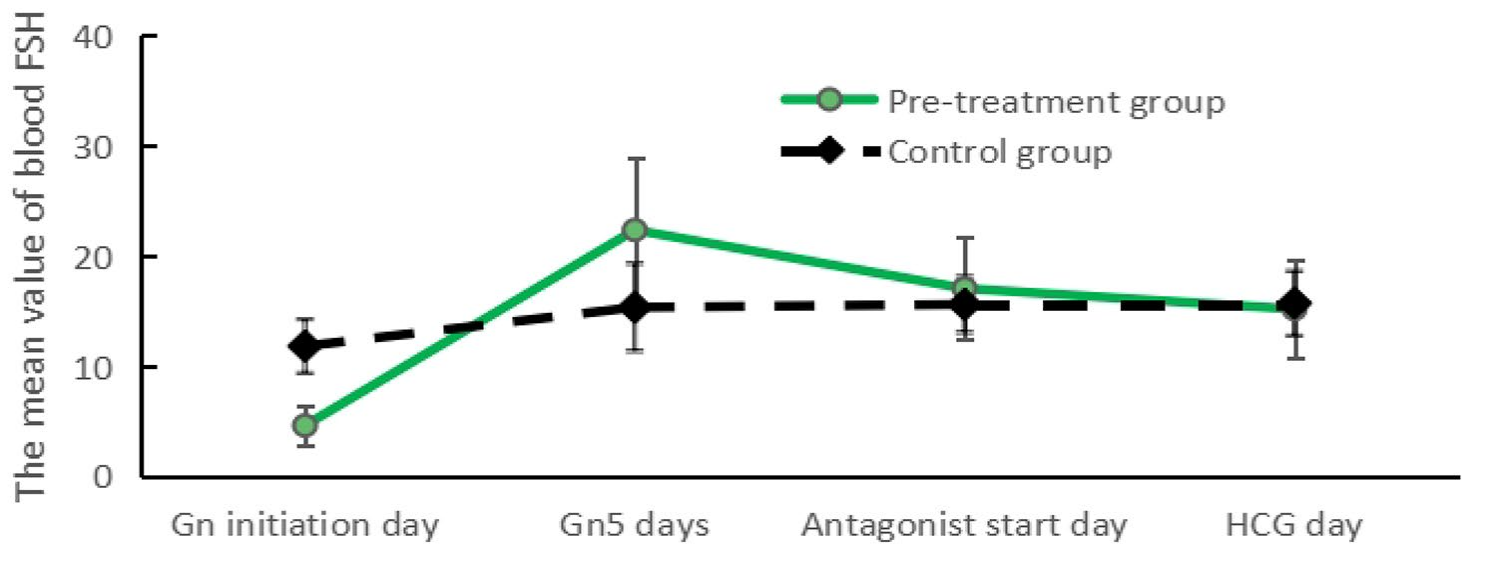
Four different time of COH

**Fig. 2:**
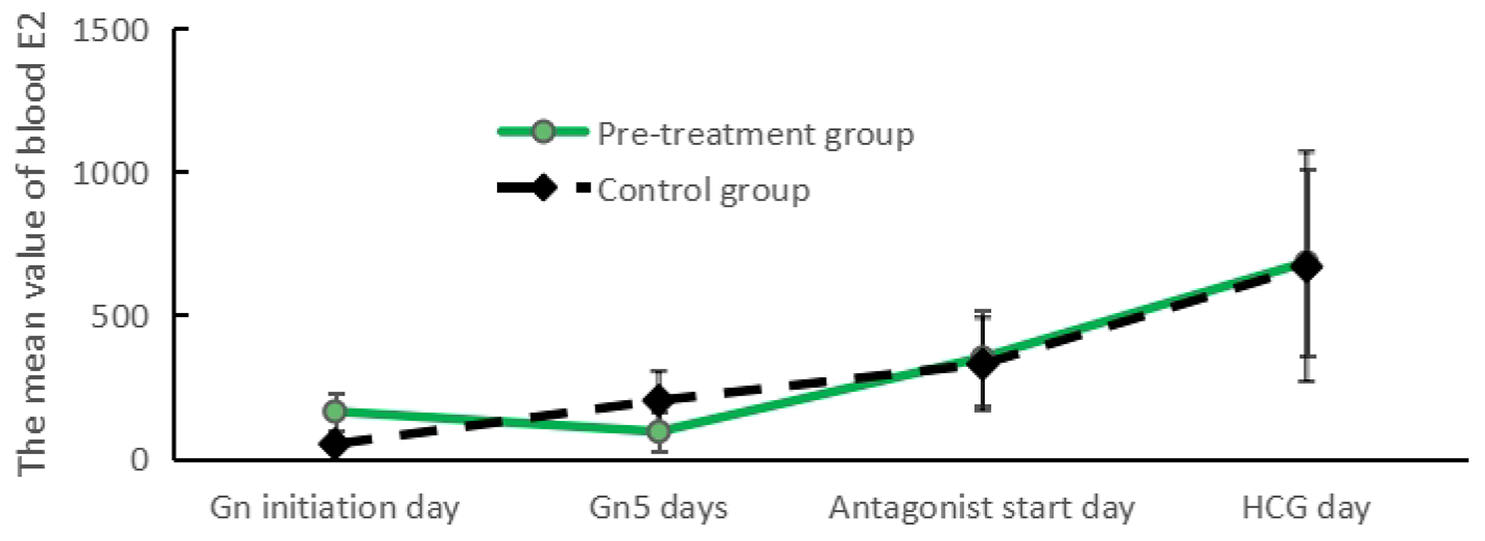
Four different time of COH

FSH levels were significantly lower and E_2_ levels were significantly higher in the Gn initiation day estrogen pretreatment group than in the control group (*P* < 0.05; Figs. 1 and 2). FSH levels increased significantly in both groups on day Gn5, with a more pronounced increase in FSH levels in the estrogen pretreatment group and a more pronounced increase in E_2_ levels in the control group. FSH levels decreased on the antagonist start day in the estrogen pretreatment group (*P* < 0.05), whereas FSH levels in the control group did not fluctuate significantly; estrogen levels in both groups were comparable (*P* > 0.05). The FSH and E_2_ levels were comparable in both groups on the day of HCG administration (*P* > 0.05).

### Laboratory indicators and pregnancy outcomes

There were no statistically significant differences in the number of retrieved oocytes, number of MII oocytes, number of 2PN fertilizations, number of 2PN egg cleavages, number of D_3_ high-quality embryos, blastocyst formation rate, high-quality blastocyst formation rate, endometrial thickness on HCG day, number of transferred embryos, or number of transferred high-quality embryos between the two groups (*P* > 0.05). There were no statistically significant differences in the clinical pregnancy rate of fresh embryos, cumulative pregnancy rate, or rate of embryos without transfer (including the percentage of follicular dysplasia) between the two groups (*P* > 0.05; Table 3).

**Table 3:** Laboratory indicators and pregnancy outcomes

	Pretreatment group (*n* = 79)	Control group(*n* = 322)	*P*-value
Retrieved oocytes (n)	2.07 ± 0.80	2.06 ± 0.88	0.909
No. of MII oocytes (n)	1.28 ± 1.14	1.16 ± 1.09	0.412
No. of 2PN fertilization (n)	0.89 ± 0.78	0.94 ± 0.86	0.518
No. of 2PN oogenesis (n)	0.89 ± 0.7	0.95 ± 0.82	0.416
No. of D3 quality embryos (n)	0.63 ± 0.40	0.57 ± 0.34	0.536
Blastocyst formation rate^a^ (%)	55.56(15/27)	46.59(82/176)	0.386
Quality blastocyst formation rate^b^ (%)	44.44(12/27)	37.5(66/176)	0.488
Endometrial thickness on HCG day (mm)	10.38 ± 2.48	9.87 ± 2.15	0.082
No. of embryos transferred (n)	1.20 ± 0.40	1.33 ± 0.47	0.120
No. of high quality embryos for transfer (n)	0.89 ± 0.61	0.84 ± 0.66	0.683
Transferred Clinical pregnancy rate of fresh embryos (%)	28.89(13/45)	34.65(35/101)	0.493
Cumulative pregnancy rate^c^ (%)	23.94(17/71)	21.05(60/285)	0.597
No transferable embryo rate^d^ (%)	34.18(27/79)	30.12(97/322)	0.483
Follicular dysplasia rate (%)	10.13(8/79)	7.14(23/322)	0.374

Note:

^a^Blastocyst formation rate = (D5/D6/total number of blastocysts)/normally fertilized number of fertilized eggs × 100 [10, 11]

^b^Quality blastocyst formation rate = number of quality blastocysts / number of normally fertilized eggs × 100 [11]

^c^Cumulative pregnancy rate = number of first pregnancies after the current egg retrieval cycle (including fresh embryo transfer and FET cycle) / number of egg retrieval cycles × 100; remaining frozen embryos that did not achieve pregnancy are not counted in the cumulative pregnancy rate

^d^No transferable embryos rate = number of cycles without transferable embryos / number of ovulation cycles × 100; no transferable embryos include no egg retrieval, no egg acquisition, no fertilization or abnormal fertilization, abnormal egg cleavage, poor quality embryos and no (high quality) blastocyst formation

## Discussion

In the late luteal phase, as the corpus luteum shrinks and estrogen and progesterone levels decrease, the inhibition of negative feedback to the pituitary gland is lifted, and FSH levels begin to rise. A portion of small follicles with a low threshold for the FSH response may begin to develop in the late luteal phase, resulting in uneven AFC size in the early follicular phase and asynchronous follicular development in the late follicular phase [12]. This affects the number of eggs obtained and embryos available for transfer, in turn affecting the outcome of the superovulatory cycle [13].

In DOR patients with elevated basal FSH levels, prolonged higher levels of FSH stimulation can lead to the downregulation of FSH receptors, making it difficult to overcome the effects of the luteal phase of the previous cycle on small follicle recruitment, even when high doses of FSH are administered in the early follicular phase. Therefore, controlling elevated FSH levels in the late luteal phase of the preceding cycle in an antagonist regimen is the key to improving follicular synchronization. Most current studies use estrogens, progestins, contraceptives (OC), and antagonists for pretreatment [14–16]. Pretreatment with estrogen, progestin, or OC can eliminate the corpus luteum, suppress endogenous FSH, restore serum FSH to normal levels, and restore the sensitivity of FSH receptors [14]. Miyoshi et al. suggested that estrogen can upregulate FSH and LH receptors on granulosa cells to increase their sensitivity to Gn and synergize with FSH to promote follicular growth and proliferation of granulosa cells [15]. Meta-analysis showed that OC combined with GnRH antagonist treatment decreased the rates of ongoing pregnancy, however there was also no evidence of a difference in live birth or clinical pregnancy rates between women who were pretreated with and without estrogen [16, 17].

Previous studies have shown that 4 mg/day of estrogen is effective in suppressing premature FSH [18, 19]. In this study, 17β-estradiol (4 mg/day) was chosen for pretreatment. We found that the estrogen pretreatment group had lower FSH levels on the initiation day, and the mean follicle diameter and maximum sinus follicle diameter were significantly smaller than those in the control group, which significantly improved follicular synchronization, in agreement with the study of Fanchin et al. [3]. This may be because estrogen pretreatment negatively inhibits the hypothalamus and pituitary gland and suppresses FSH levels, which in turn inhibits follicle growth. The lower estradiol levels, smaller mean follicle diameter, and smaller maximum follicle diameter in the pretreatment group on day 5 were mainly because the follicle diameter was already smaller at the time of initiation. This may explain the larger total Gn and longer Gn-days in this study, which is in agreement with previous studies [18]. Chang E M et al. concluded that estrogen pretreatment in patients with poor ovarian response significantly increases the number of eggs gained and effective embryos, which may reduce cancelled cycles [20]. In this study, FORT was used to supplement follicular synchronization. The results showed no difference in FORT between the two groups, further indicating that estrogen pretreatment did not increase follicular synchronization. We concluded that in the elevated basal FSH combined with DOR population, estrogen pretreatment with an antagonist regimen does not increase the number of eggs gained, the number of MII eggs, the number of good quality embryos, or the clinical and cumulative pregnancy rates, but does results in a longer Gn time and increased dosage, similar to the results of the studies by Zhang et al. [21] as well as Mutlu MF et al. [22]. In fact, the DOR population in our study was even worse.This is because the mean number of oocytes retrieved in our study was 2 compared to 3 in Zhang et al. and close to 4 in Mutlu MF et al. There are no effective treatments to improve the prognosis for severe cases of DOR.

Serum FSH on days 2–3 of menstruation are often used as important indicators for evaluating ovarian reserve function. Basal FSH of ≥ 10 U/L are found in patients with poor pregnancy outcome [5]. It has been suggested that patients with elevated basal FSH levels have an increased risk of ovarian hyporesponsiveness [22]. Luna et al. suggested that elevated basal FSH levels at < 35 years of age indicate reduced ovarian responsiveness to Gn [23]. Tartagni et al. found that in women with premature ovarian failure, follicles began to develop when FSH levels were reduced to 15 IU/L with estrogen .When the endogenous FSH level dropped below 15 IU/L, IVF/ICSI treatment was significantly improved by the administration of ovulation promotion [24]. In the estrogen-pretreated group in this study, the FSH level was significantly higher than that in the control group on day 5 of Gn treatment, with a mean level of 22.34 U/L. It may be that after discontinuation of estrogen, negative feedback to the hypothalamic-pituitary-ovarian axis was released, exogenous FSH combined with endogenous FSH increased, and the excessive FSH led to reduced ovarian responsiveness to Gn and more patients with poorly cancelled cycles due to follicular development. Ashrafi et al. concluded that increasing the duration of the E_2_ pretreatment after the onset of menses and delaying the start of the gonadotrophins in patients with poor ovarian response improved fertilization rates, embryo quality, and cycle cancellation rates [25]. It has be shown in normoresponders to increase basal LH levels by positive feed back [26]. This could lead in DOR to increase local ovarian androgens and up regulate FSH receptors. In our study, the follicular output rate, MII egg count, D_3_ quality embryo count, blastocyst formation rate, clinical pregnancy rate, cumulative pregnancy rate, and cycle cancellation rate were similar in both groups. This indicates that estrogen pretreatment did not increase egg acquisition or improve clinical outcomes.

Our fertility center is one of the first in China to use antagonist-led superovulation protocols. Luteal-phase estrogen pretreatment is routinely used for patients with normal and low ovarian reserves. During the course of treatment, it was found that more patients with elevated basal FSH levels combined with DOR had their cycles cancelled at Gn5 because of poor follicle growth after pretreatment with estrogen during the first treatment cycle. This led to more patients choosing the antagonist regimen without pretreatment in later stages. This is the main reason for the difference in sample size between the two groups in this study.Our retrospective study provides novel results that need to be explored and confirmed in further prospective research efforts.

## Conclusions

The use of luteal phase estrogen pretreatment for patients with elevated basal FSH combined with DOR resulted in high FSH levels after the release of negative feedback. This was detrimental to early follicular growth, did not increase follicular output rates, may have increased the use and duration of controlled ovarian stimulation drugs, and did not increase the number of eggs gained or improve clinical outcomes.

## Data Availability

The datasets that were used and/or analysed during the current study are available from the corresponding author upon reasonable request.
